# Data Versus Truth in the Midst of the COVID-19 Pandemic

**DOI:** 10.1177/2333392820970681

**Published:** 2020-11-05

**Authors:** Jim Rohrer

**Affiliations:** 1 Walden University, Minneapolis, MN, USA

Perhaps at no time in history has more data about a global pandemic been so rapidly and freely available. Anyone with a computer can download current data and analyze it independently. Several forecasting models have been developed and their differing projections are easily found on government websites. Uncounted scientific articles have been published about the pandemic.

Missing from all this information and analysis is frank recognition of the uncertainty in the assumptions upon which data analysis and forecasts are based. State mitigation strategies are based partly on guidance from the Centers for Disease Control and Prevention (CDC) and partly on local politics. The effectiveness of different mitigation strategies is not strongly supported by population-based evidence, yet television news programs constantly bring out ‘experts’ who insist that if we only did this or that, pandemic deaths would have been avoided.

**Figure 1.**
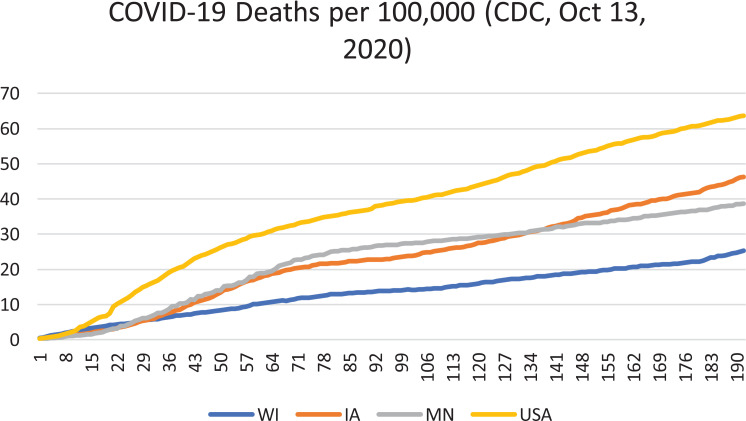
Trends in COVID-19 deaths per 100,000 in selected states.

Consider the situation in three contiguous states: Iowa, Minnesota, and Wisconsin. Figure 1 shows the latest data from CDC on deaths per 100,000 in those three states compared to the United States as a whole. The first conclusion we reach is that all three states have lower deaths per 100,000 than the US. Wisconsin is the lowest, but a recent surge in cases might cause that line to shift above Minnesota and Iowa. We can only wait and see what happens. However, if Wisconsin moves upward, that will make their trend line even closer to the lines for Minnesota and Iowa. In sum, we might argue that the three states are more similar to each other than they are to the US average.

However, the governors of these states have followed different mitigation strategies. Iowa has been the least restrictive of the three. Closures occurred at different rates in different localities. State policy now is to be reopened. In contrast, Minnesota moved more aggressively toward locking down the state and was more cautious about reopening. Wisconsin seems to have been a mixture. They reopened the state but have imposed new restrictions. The politics of the three states explain these differences in policy. Iowa was a GOP state in 2016. Minnesota seems firmly in the blue-state column. Wisconsin is generally classed as a battleground state.

Despite their differences in mitigation policy, deaths per hundred thousand in all three states have been lower than the national rate. Why might this be so? Some demographic information is presented in the Table 1. In general, we can safely say that all three states are less urban than the US overall, have lower population densities, and have total populations that are modest. Demographically, they are similar. Quantifying the specific effects of demographic variables on COVID-19 deaths per 100,000 is not yet possible. However, we might wonder if demographics have more to do with pandemic mortality than state government policy.

**Table 1.** Selected Demographic Variables in Iowa, Minnesota and Wisconsin.

	Total Population (Millions)	Population Density (Per Square Mile)	Percent Urban
Iowa	3.16	56.5	64.0
Minnesota	5.64	70.8	73.3
Wisconsin	5.82	107.5	70.2

Why might state policies not be as effective in practice than they are in theory? As researchers, we should know that policies based on studies in laboratory conditions lack external validity and may not be as effective in the real-world. People in the community do not follow strict protocols and may not pay attention to regulations. In fact, regulations generate defiance. Furthermore, small laboratory studies do not reveal the negative consequences of mitigation policies. A search of the scientific literature on the impact of lockdowns reveals more articles about harms than benefits.

Perhaps the most serious source of bias in the media is reliance on practicing clinicians as experts even if they lack any special expertise in population epidemiology. These experts typically are working in hospitals and they are over-burdened with acute COVID-19 cases. They see the most biased sample possible and this influences their opinions.

Finally, we should recognize that medical specialization affects policy recommendations. COVID-19 is a highly contagious respiratory disease. It is not a sexually transmitted disease (STD) like HIV. Mitigation strategies used for STDs may not be effective for COVID-19. After all, this virus could be contracted when someone sneezes in the next aisle of the grocery store. We simply cannot track all exposures. Yet, our mitigation recommendations seem to be coming from experts whose experience is in STDs rather than respiratory epidemiology.

As researchers, we should be concerned with the false and misleading interpretations of the available data that fill the airwaves. Instead of freely admitting uncertainties about the effectiveness of government policies, we hear arguments that the national failure to follow certain policies has caused many deaths. We do not know to what extent that assertion is true; we cannot say how much the harms of lockdowns counter the benefits. Let me suggest that responsible researchers will challenge the assertions based on weak data by saying we do not yet know how much effect different policies would have had in the US.

